# Hard Ride: Traffic-Related Pollution May Alter Heart Function in Urban Cyclists

**DOI:** 10.1289/ehp.119-a443a

**Published:** 2011-10-01

**Authors:** Julia R. Barrett

**Affiliations:** Julia R. Barrett, MS, ELS, a Madison, WI–based science writer and editor, has written for *EHP* since 1996. She is a member of the National Association of Science Writers and the Board of Editors in the Life Sciences.

People who ride bicycles along roadways may incur considerable exposure to traffic-related air pollutants that have been associated with adverse respiratory and cardiovascular effects in epidemiologic studies. A new study examined changes in heart rate variability (HRV) and respiratory factors in cyclists exposed to traffic-related air pollutants and found that ultrafine particles as well as ozone and nitrogen dioxide (NO_2_) altered autonomic regulation of the heart [*EHP* 119(10):1373–1378; Weichenthal et al.].

Forty-two healthy individuals (28 men and 14 women aged 19–58 years) participated in the study, which involved cycling for 1 hour indoors or along high-traffic or low-traffic routes in Ottawa, Ontario. Thirty-eight participants completed all three routes. Continuously recorded electrocardiograms provided cardiac data, and respiratory function was measured by spirometry. Cyclists were equipped with pannier-mounted instruments to collect real-time information on fine particles, ultrafine particles, and black carbon, while technicians on bicycles equipped with volatile organic compound monitors traveled with the participants. Data for ambient ozone, NO_2_, and sulfur dioxide came from a fixed monitoring station in downtown Ottawa.

In the 3 hours after cycling, short-term exposure to traffic-related air pollution was associated with altered autonomic regulation of the heart, specifically parasympathetic modulation. No strong effects on respiratory measures were observed.

**Figure:**
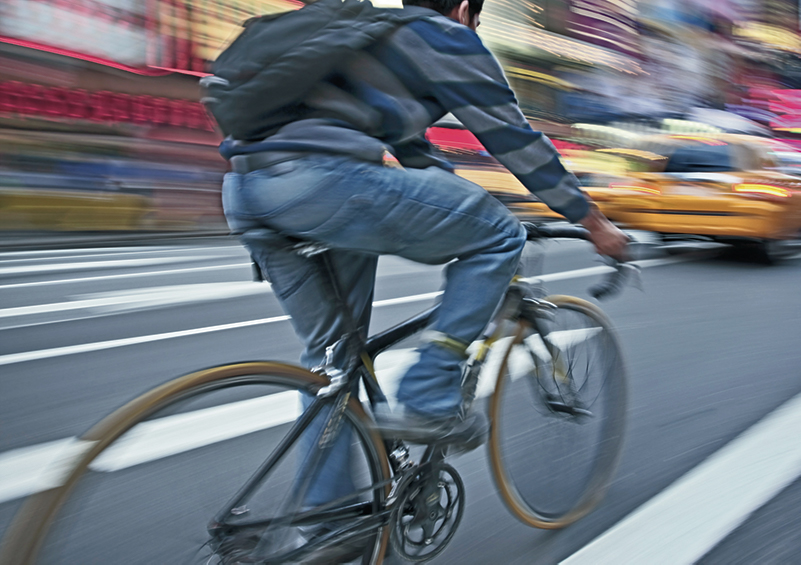
Exposure to traffic-related pollutants during cycling could pose a threat to certain vulnerable riders. © SVLuma/Shutterstock

The study was strengthened by using personal exposure measures, real-life conditions, and a cross-over design, but it was potentially limited by the lack of data on exposures encountered by participants en route to the study sites, an inability to adjust for respiratory effects on HRV, the small sample size, the short time frame of post-cycling measurements, and the exclusion of additional cardiovascular measures. Additionally, individual exposure data were unavailable for NO_2_ and ozone, and associated effects may have been underestimated.

The health benefits of cycling might outweigh the impact of traffic-related air pollution for healthy individuals. However, the effects on cardiac autonomic function demonstrated in this study could potentially be harmful in individuals with underlying cardiovascular issues. Consequently, the authors suggest that bicycle routes and paths be planned so as to avoid exposure to motor vehicle traffic.

